# Construction of a Prediction Model for the Mortality of Elderly Patients with Diabetic Nephropathy

**DOI:** 10.1155/2022/5724050

**Published:** 2022-09-12

**Authors:** Li Wang, Yan Lv

**Affiliations:** ^1^Geriatrics Department of Shenzhen Luohu People's Hospital, Shenzhen 518000, Guangdong, China; ^2^Department of Nephrology, Shanxi Bethune Hospital, Shanxi Academy of Medical Sciences, Tongji Shanxi Hospital, Third Hospital of Shanxi Medical University, Taiyuan 030032, Shanxi, China

## Abstract

To construct a prediction model for all-cause mortality in elderly diabetic nephropathy (DN) patients, in this cohort study, the data of 511 DN patients aged ≥65 years were collected and the participants were divided into the training set (*n* = 358) and the testing set (*n* = 153). The median survival time of all participants was 2 years. The data in the training set were grouped into the survival group (*n* = 203) or the death group (*n* = 155). Variables with *P* ≤ 0.1 between the two groups were selected as preliminary predictors and involved into the multivariable logistic regression model and the covariables were gradually adjusted. The receiver operator characteristic (ROC), Kolmogorov-Smirnov (KS), and calibration curves were plotted for evaluating the predictive performance of the model. Internal validation of the performance of the model was verified in the testing set. The predictive values of the model were also conducted in terms of people with different genders and ages or accompanied with chronic kidney disease (CKD) or cardiovascular diseases (CVD), respectively. In total, 216 (42.27%) elderly DN patients were dead within 2 years. The prediction model for the 2-year mortality of elderly patients with DN was established based on length of stay (LOS), temperature, heart rate, peripheral oxygen saturation (SpO_2_), serum creatinine (Scr), red cell distribution width (RDW), the simplified acute physiology score-II (SAPS-II), hyperlipidemia, and the Chronic Kidney Disease Epidemiology Collaboration equation for estimated glomerular filtration rate (eGFR-CKD-EPI). The AUC of the model was 0.78 (95% CI: 0.73–0.83) in the training set and 0.72 (95% CI: 0.63–0.80) in the testing set. The AUC of the model was 0.78 (95% CI: 0.65–0.91) in females and 0.78 (95%CI: 0.68–0.88) in patients ≤75 years. The AUC of the model was 0.74 (95% CI: 0.64–0.84) in patients accompanied with CKD. The model had good predictive value for the mortality of elderly patients with DN within 2 years. In addition, the model showed good predictive values for female DN patients, DN patients ≤75 years, and DN patients accompanied with CKD.

## 1. Introduction

Diabetic nephropathy (DN) is a common microvascular complication of diabetes mellitus (DM) [1]. Approximately 30% of DM patients are diagnosed with renal complications including DN [2]. DN in patients can lead to end‐stage renal failure and disability, which is associated with high mortality all over the world [3]. DN patients tend to be elderly and may be associated with various complications, such as cerebrovascular, cardiovascular, peripheral vascular, connective tissue, liver, and chronic pulmonary diseases and tumors [4, 5]. DN is associated with higher mortality rates and worse clinical outcomes, which were largely due to the serious complications [6]. Therefore, predicting the all-cause mortality in DN patients was of great value for providing timely interventions in these patients and improving the outcomes of these patients.

Previously, various studies have explored the risk factors for the mortality in DN patients [7–9], but the risk of mortality could not be estimated based on the findings of these studies, as they did not form a prediction model. Currently, the model for predicting the mortality of DN patients was rare. In 2017, Sato et al. [10] established a prediction model for all-cause mortality in DN patients [10]. The model had an area under the curve (AUC) of 0.791, which had good predictive ability for the mortality of DN patients. Previously, multiple studies have indicated that prediction model based on combined variables might be better than those including only one variable [11]. The prediction model by Sato et al. [10] was focused on predialysis neutrophil-lymphocyte ratio, and validation was not performed to verify the performance of the model. Due to the poor prognosis of DN patients at old age [12], a suitable prediction model was required for the all-cause mortality in elderly DN patients to quickly identify those at high risk of mortality and provide timely treatments for these patients.

In this study, the purpose was to construct a prediction model for all-cause mortality in elderly DN patients. The predictors were screened out and included in the model. The internal validation was performed to evaluate the predictive value of the model. Subgroup analysis was also conducted in terms of gender and being complicated with chronic kidney disease (CKD) or cardiovascular diseases (CVD).

## 2. Methods

### 2.1. Study Population

In this cohort study, the data of 522 DN patients aged ≥65 years were derived from Medical Information Mart for Intensive Care (MIMIC-III) database. MIMIC-III database is an extensive and single-center database, constructed by Institutional Review Boards (IRB) of the Massachusetts Institute of Technology (Cambridge, MA, USA) and Beth Israel Deaconess Medical Center. It contained the data of over 50000 hospital patients admitted to intensive care units (ICUs) between 2001 and 2012 including the demographic details, admission and discharge times, dates of death, procedures such as dialysis, imaging studies, blood chemistry, hematology, urine analysis, microbiology test results, administration records of intravenous medications, medication orders, free text notes such as provider progress notes and hospital discharge summaries, and nurse-verified vital signs [13]. After excluding participants without the data on Sequential Organ Failure Assessment (SOFA) score, the simplified acute physiology score-II (SAPS-II), and temperature, 511 patients were finally involved in our study.

### 2.2. Potential Predictors

Potential predictors were analyzed in this study including gender, marital status (divorced, married, separated, single, widowed, or unknown), ethnicity (Asian, Black, Hispanic or Latino, White, others, or unknown), length of stay (LOS, day), age (years), respiratory rate (times/min), temperature (°C), heart rate (times/min), systolic blood pressure (SBP, mmHg), diastolic blood pressure (DBP, mmHg), mean arterial pressure (MAP, mmHg), peripheral oxygen saturation (SpO_2_, %), white blood cells (WBC, 10^3^/*μ*L), red blood cells (RBC, 10^3^/*μ*L), sodium (mEq/L), potassium (mEq/L), phosphate (mEq/L), calcium (mEq/L), magnesium (mEq/L), platelets (PLT, k/*μ*L), lactate, international normalized ratio (INR), mean corpuscular volume (MCV, fl), glucose (mg/dL), serum creatinine (Scr, mg/dL), blood urea nitrogen (BUN, mg/dL), bicarbonate, hematocrit, hemoglobin, mean corpuscular hemoglobin concentration (MCHC, 10 g/L), red cell distribution width (RDW, %), chronic obstructive pulmonary disease (COPD, no or yes), atrial fibrillation (AF, no or yes), liver cirrhosis (no or yes), respiratory failure (no or yes), hyperlipidemia (no or yes), malignant cancer (no or yes), SAPS-II, SOFA score, insulin (no or yes), metformin (no or yes), survival time, the Chronic Kidney Disease Epidemiology Collaboration equation for estimated glomerular filtration rate (eGFR-CKD-EPI, mL/min/m^2^), the Modification of Diet in Renal Disease equation for estimated glomerular filtration rate (eGFR-MDRD, mL/min/m^2^), CVD (no or yes), CKD (no or yes), myocardial infarction (no or yes), hypertension (no or yes), and peripheral vascular disease (no or yes).

### 2.3. Outcome Variables

The outcome variable was the death of elderly DN patients within 2 years. The follow-up time was 10 years and the median survival time was 2 years.

### 2.4. Definitions of Variables

eGFR-MDRD = 175.0 × Scr  −1.154 × age−0.203 × 0.742 (if female) × 1.212 (if black); eGFR-CKD-EPI = 141 × min (Scr/*κ*, 1) *α* × max (Scr/*κ*, 1) − 1.029 × 0.993 age × 1.108 (if female) × 1.159 (if black). *κ* is 0.7 for females and 0.9 for males, *α* is −0.329 for females and −0.411 for males, min indicates the minimum of Scr/*κ* or 1, and max indicates the maximum of Scr/*κ* or 1. LOS is the length of stay in the ICUs.

### 2.5. Logistic Regression Model

Logistic regression is a classification method applied for binary or classification method generalizing logistic regression to multiclass problems multinomial outcome variables. It evaluates the associations between a dependent categorical outcome and one or more independent predictor variables, which provides predicted probabilities for each category [14] (1). The detailed formula of the logistic regression model is as follows:

(1) $$\begin{array}{c} \log\;it\;P=\ln\left(\frac{P}{1-P}\right)=a+b_{1}x_{1}+b_{2}x_{2}+\cdots +b_{m}x_{m},\\ P=\frac{e^{a+b_{1}x_{1}+b_{2}x_{2}+\cdots +b_{m}x_{m}}}{1+e^{a+b_{1}x_{1}+b_{2}x_{2}+\cdots +b_{m}x_{m}}}. \end{array}$$

### 2.6. Statistical Analysis

The normal distributed measurement data were expressed as mean ± standard deviation (mean ± SD), and comparisons between groups were subjected to independent-sample *t*-test. Nonnormal distributed data were described as *M* (*Q*_1_, *Q*_3_), and the Mann-Whitney *U* rank-sum test was used for comparing differences between groups. The enumeration data were displayed as *n* (%), and comparisons between groups were performed by *χ*^2^ test or Fisher's exact probability method [15]. All the data were divided into the training set (*n* = 358) and the testing set (*n* = 153) at a ratio of 7 : 3^16^. The prediction model was constructed in the training set and verified in the testing set. The data in the training set were grouped into the survival group (*n* = 203) or the death group (*n* = 155), and comparisons between the two groups were performed. Variables with *P* ≤ 0.1 were selected as preliminary predictors. The preliminarily screened predictors were then involved in the multivariable logistic regression model and the covariables were gradually adjusted. Subgroup analysis was conducted in male group and female group, CKD group and non-CKD group, CVD group and non-CVD group, age ≤75 years group, and age >75 years group, respectively. The area under the curve (AUC), Kolmogorov-Smirnov (KS), calibration curve, sensitivity, specificity, negative predictive value (NPV), positive predictive value (PPV), and accuracy were employed for evaluating the predictive performance of the model. A nomogram was also plotted to evaluate the possibility of mortality of elderly patients with DN. The confidence level was 0.05 and Python 3 was used for statistical analysis.

## 3. Results

### 3.1. Missing Value Manipulation and Sensitivity Analysis

The missing values of variables are shown in Supplementary Table 1. The missing data were manipulated via multiple interpolation using *R* mice. Sensitivity analysis was performed in the data before and after the manipulation. The results delineated that there was no statistical difference between the data before and after the manipulation, indicating that the data after manipulation could be used for further analysis.

### 3.2. Baseline Characteristics of Participants

**Figure 1:**
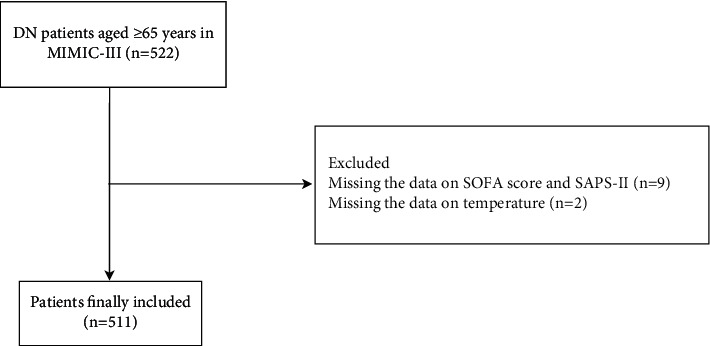
The screen process of the participants.

In total, 522 DN patients aged ≥65 years from MIMIC-III were involved in our study. Participants without the data on SOFA score and SAPS-II (*n* = 9) and those without the data on temperature (*n* = 2) were excluded, and 511 patients were finally included. The detailed screen process is shown in Figure 1. Among them, 292 people were males, accounting for 57.14%. The median LOS was 2.6 days. The median age of all participants was 74.39 years. The median glucose level was 166 mg/dL. The median Scr level was 2.7 mg/dL. The median BUN was 45 mg/dL. The median survival time of all patients was 652.00 days. The median eGFR-CKD-EPI was 21.44 mL/min/m^2^ and the median eGFR-MDRD was 21.8 mL/min/m^2^. There were 389 patients accompanied with CVD, accounting for 76.13%, and 333 patients accompanied with CKD, accounting for 65.17%. The median survival time of all participants was 730 days and 216 people died within 2 years, accounting for 42.27%. The LOS in the survival group was shorter than that in the death group (2.15 days versus 3.24 days). The median survival time of the participants in the survival group was longer than that in the death group (730.00 days versus 61.50 days) (Table 1). The equilibrium test revealed that there was no significant difference between the data of participants in the training set and the testing set (Table 2).

LOS: length of stay, SBP: systolic blood pressure, DBP: diastolic blood pressure, MAP: mean arterial pressure, SpO_2_: peripheral oxygen saturation, WBC: white blood cells, RBC: red blood cells, INR: international normalized ratio, MCV: mean corpuscular volume, MCHC: mean corpuscular hemoglobin concentration, RDW: red cell distribution width, COPD: chronic obstructive pulmonary disease, AF: atrial fibrillation, eGFR-CKD-EPI: the Chronic Kidney Disease Epidemiology Collaboration equation for estimated glomerular filtration rate, eGFR-MDRD: the Modification of Diet in Renal Disease equation for estimated glomerular filtration rate, CKD: chronic kidney disease, CVD: cardiovascular diseases, SOFA: Sequential Organ Failure Assessment, SAPS-II: the simplified acute physiology score-II.

### 3.3. Comparisons between the Characteristics of Patients in the Survival Group and Death Group in the Training Set

The median LOS (2.15 days versus 3.01 days, *Z* = 3.734), age (73.59 years versus 76.03 years, *Z* = 1.770), INR (1.20 versus 1.30, *Z* = 2.767), Scr (2.30 mg/dL versus 2.90 mg/dL, *Z* = 2.100), BUN (43.00 mg/dL versus 50.00 mg/dL, *Z* = 2.447), SOFA score (5.00 versus 6.00, *Z* = 4.397), the average heart rate (80.12 times/min versus 85.81 times/min, *t* = −2.95), SpO_2_ (96.52 versus 97.42, *t* = −1.77), RBC (3.72 10^3^/*μ*L versus 3.58 10^3^/*μ*L, *t* = 1.85), SAPS-II (40.28 versus 45.77, *t* = −4.62), and the proportion of patients with respiratory failure (23.65% versus 33.55%, *χ*^2^ = 4.282) were lower in the survival group than in the death group. The median eGFR-MDRD (25.42 mL/min/m^2^ versus 20.41 mL/min/m^2^, *Z* = −2.266), eGFR-CKD-EPI (25.60 mL/min/m^2^ versus 19.68 mL/min/m^2^, *Z* = −2.705), the average temperature (36.59°C versus 36.41°C, *t* = 1.75), calcium (8.82 mEq/L versus 8.57 mEq/L, *t* = 2.48), hemoglobin (11.02 versus 10.61 *t* = 2.03), MCHC (33.05 10 g/L versus 32.59 10 g/L, *t* = 2.78), and the proration of patients with hyperlipidemia (60.59% versus 34.19%, *χ*^2^ = 4.282), CKD (70.44% versus 60.65%, *χ*^2^ = 3.771), diabetic retinopathy (21.18% versus 12.26%, *χ*^2^ = 4.888), and insulin use (94.58% versus 89.68%, *χ*^2^ = 3.031) in the survival group were higher than those in the death group. The proportion of patients with different marital status was statistically different between the survival group and the death group (*χ*^2^ = 10.722) (Table 3).

### 3.4. Predictors for Mortality of Elderly Patients with DN

Variables with *P* ≤ 0.1 in the survival group and the death group were included in the multivariable logistical analysis. Stepwise regression was applied to identify the predictors for mortality of elderly patients with DN within 2 years. As depicted in Table 4, LOS (OR = 1.10, 95% CI: 1.03–1.17), temperature (OR = 0.74, 95% CI: 0.63–0.88), heart rate (OR = 1.03, 95% CI: 1.01–1.04), SpO_2_ (OR = 1.06, 95% CI: 1.01–1.11), Scr (OR = 0.83, 95% CI: 0.69–0.98), RDW (OR = 1.25, 95% CI: 1.10–1.42), SAPS-II (OR = 1.02, 95% CI: 1.01–1.05), hyperlipidemia (OR = 0.43, 95% CI: 0.27–0.70), and eGFR-CKD-EPI (OR = 0.97, 95% CI: 0.94–0.99) were predictors associated with the risk of mortality in elderly patients with DN within 2 years. The final model was Log (*p*/1 − *p*) = 0.09 × LOS −  0.29 × temperature − 0.19 ×  creatinine + 0.03 × heart rate + 0.05 × SpO_2_ + 0.22 × RDW +  0.02 × SAPS-II-0.84 × hyperlipidemia − 0.03 × eGFR-CKD-EPI.

### 3.5. Predictive Value of the Model

**Figure 2:**
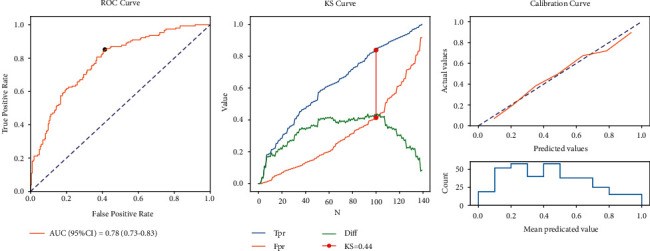
The AUC, KS, and calibration curves of the model in the training set.

**Figure 3:**
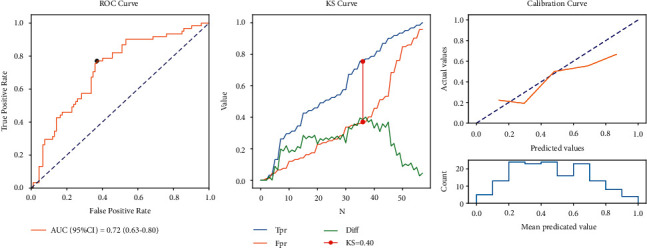
The AUC, KS, and calibration curves of the model in the testing set.

**Figure 4:**
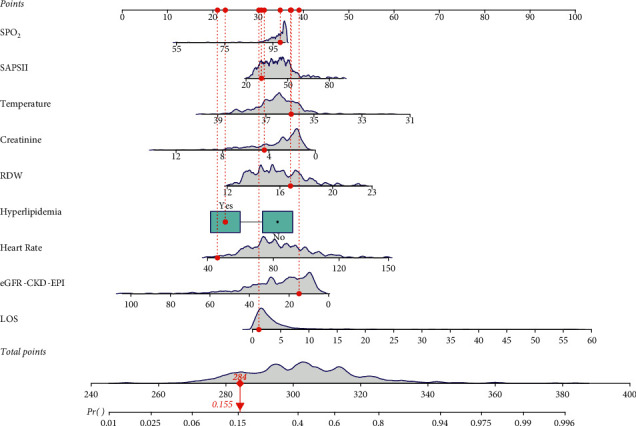
The nomogram of the prediction model.

According to the data in Table 5, for the model in the training set, the sensitivity was 0.85 (95% CI: 0.80–0.91), the specificity was 0.59 (95% CI: 0.52–0.65), the PPV was 0.61 (95% CI: 0.55–0.68), the NPV was 0.84 (95% CI: 0.78–0.90), the AUC was 0.78 (95% CI: 0.73–0.83), and the accuracy was 0.70 (95% CI: 0.65–0.75). The ROC, KS, and calibration curves in the training set are shown in Figure 2. For the model in the testing set, the sensitivity was 0.90 (95% CI: 0.83–0.98), the specificity was 0.47 (95% CI: 0.37–0.57), the PPV was 0.53 (95% CI: 0.43–0.62), the NPV was 0.88 (95% CI: 0.79–0.97), the AUC was 0.72 (95% CI: 0.63–0.80), the accuracy was 0.64 (95% CI: 0.56–0.72). The ROC, KS, and calibration curves in the testing set are exhibited in Figure 3. The nomogram was plotted and a sample was selected, which showed that the total score of the patient was 284, and the predicted mortality probability was 0.155, which was lower than the cut-off, 0.33 (Figure 4). The predicted outcome of the patient was survival, which was consistent with the actual outcome.

### 3.6. The Predictive Value of the Model concerning Different Subgroups

#### 3.6.1. Gender

In the male group, the sensitivity was 0.90 (95% CI: 0.80–0.99), the specificity was 0.39 (95% CI: 0.25–0.52), the PPV was 0.54 (95% CI: 0.42–0.66), the NPV was 0.83 (95% CI: 0.67–0.98), the AUC was 0.66 (95% CI: 0.55–0.78), and the accuracy was 0.61 (95% CI: 0.51–0.72). In the female group, the sensitivity was 0.91 (95% CI: 0.79–1.00), the specificity was 0.56 (95% CI: 0.41–0.71), the PPV was 0.51 (95% CI: 0.36–0.67), the NPV was 0.92 (95% CI: 0.82–1.00), the AUC was 0.78 (95% CI: 0.65–0.91), and the accuracy was 0.68 (95% CI: 0.56–0.79) (Table 6).

#### 3.6.2. Age

In patients >75 years group, the sensitivity was 0.88 (95% CI: 0.75–1.00), the specificity was 0.36 (95% CI: 0.22–0.50), the PPV was 0.43 (95% CI: 0.30–0.57), the NPV was 0.84 (95% CI: 0.68–1.00), the AUC was 0.65 (95% CI: 0.52–0.78), and the accuracy was 0.54 (95% CI: 0.43–0.66). In patients ≤75 years group, the sensitivity was 0.92 (95% CI: 0.83–1.00), the specificity was 0.57 (95% CI: 0.43–0.72), the PPV was 0.62 (95% CI: 0.49–0.75), the NPV was 0.90 (95% CI: 0.79–1.00), the AUC was 0.78 (95% CI: 0.68–0.88), and the accuracy was 0.72 (95% CI: 0.63–0.82) (Table 6).

#### 3.6.3. Accompanied with CKD or Not

In patients accompanied with CKD group, the sensitivity was 0.90 (95% CI: 0.80–1.00), the specificity was 0.51 (95% CI: 0.39–0.63), the PPV was 0.47 (95% CI: 0.34–0.59), the NPV was 0.92 (95% CI: 0.83–1.00), the AUC was 0.74 (95% CI: 0.64–0.84), and the accuracy was 0.64 (95% CI: 0.54–0.73). In patients not complicated with CKD group, the sensitivity was 0.90 (95% CI: 0.79–1.00), the specificity was 0.37 (95% CI: 0.19–0.55), the PPV was 0.61 (95% CI: 0.47–0.76), the NPV was 0.77 (95% CI: 0.54–1.00), the AUC was 0.67 (95% CI: 0.52–0.82), and the accuracy was 0.65 (95% CI: 0.53–0.77) (Table 6).

#### 3.6.4. Accompanied with CVD or Not

In patients accompanied with CVD group, the sensitivity was 0.90 (95% CI: 0.82–0.98), the specificity was 0.46 (95% CI: 0.34–0.58), the PPV was 0.57 (95% CI: 0.46–0.67), the NPV was 0.86 (95% CI: 0.75–0.97), the AUC was 0.71 (95% CI: 0.61–0.80), and the accuracy was 0.66 (95% CI: 0.57–0.74). In patients not accompanied with CVD group, the sensitivity was 0.89 (95% CI: 0.68–1.00), the specificity was 0.48 (95% CI: 0.28–0.68), the PPV was 0.38 (95% CI: 0.17–0.59), the NPV was 0.92 (95% CI: 0.78–1.00), the AUC was 0.71 (95% CI: 0.50–0.92), and the accuracy was 0.59 (95% CI: 0.42–0.75) (Table 6).

**Figure 5:**
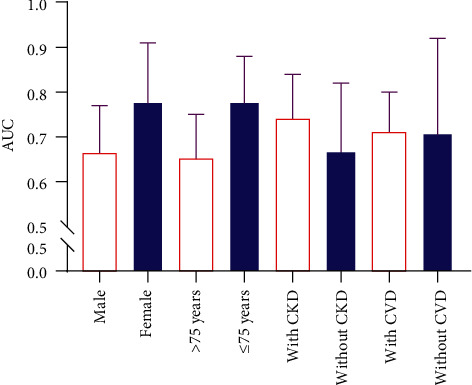
The comparisons of the AUCs of the model for different subgroups.

The comparisons of the AUCs of different subgroups delineated that the model had good predictive values for female DN patients, DN patients ≤75 years, and DN patients accompanied with CKD. The predictive values of the model for DN patients accompanied with CVD and DN patients not accompanied with CVD were similar (Figure 5).

## 4. Discussion

This study extracted the data of 511 DN patients aged ≥65 years and screened the predictors to establish a prediction model for the mortality of DN patients within 2 years. The results revealed that the model had good predictive ability for the mortality of DN patients within 2 years. Additionally, the predictive values of female DN patients, DN patients ≤75 years, DN patients accompanied with CKD, and patients with or without CVD were also good. The findings of our study might offer a tool for identifying DN patients with high risk of death within 2 years and the clinicians should provide timely interventions to those patients to improve their outcomes.

This study established a prediction model for the mortality of elderly DN patients within 2 years. In previous prediction models for the mortality of DN patents, many studies were focused on evaluating the risk of renal survival in DN patients [9, 16].Our study constructed a model and evaluated its predictive value for all-cause mortality in DN patients. DN patients were associated with various complications and the all-cause mortality of DN patients was high and should be brought to attention [17]. Sato et al. [10] established a prediction model for all-cause mortality in DN patients, but this model was based on only one laboratory index (predialysis neutrophil-lymphocyte ratio) and the sample size was small (*n* = 78). In addition, internal validation was also not performed to verify the performance of the model [10]. In our study, the prediction model was constructed based on the predictors including LOS, temperature, heart rate, SpO_2_, Scr, RDW, the simplified acute physiology score-II (SAPS-II), hyperlipidemia, and eGFR-CKD-EPI, which presented a better predictive ability compared to the model involving one predictor. The sample size in this study was larger than that in the previous study. Additionally, internal validation was performed and it was found that the predictive value of the model for the mortality of DN patents within 2 years was good. The prediction model in our study might provide a tool for the clinicians for quickly identifying DN patients with high risk of death and timely interventions should be provided in those patients for improving their outcomes. We also plotted a nomogram of the prediction model based on the results from the logistic regression. The nomogram can quickly and intuitively obtain the probability of mortality of each patient. Meanwhile, subgroup analysis was also conducted to evaluate the predictive values for patients with different gender, age, being accompanied with CKD or not, and being accompanied with CVD or not. The results revealed that the model had better predictive values for female DN patients, DN patients ≤75 years, and DN patients accompanied with CKD. The predictive values of the model for DN patients accompanied with CVD and DN patients not accompanied with CVD were similar. This indicated that the model might be more suitable for female DN patients, DN patients ≤75 years, and DN patients accompanied with CKD. These results suggested that the model could benefit specific patients with DN.

The impaired glomerular filtration rate (GFR) was regarded as a marker of DN in DM patients [18]. A previous meta-analysis revealed that the impaired GFR was an independent risk factor for progressive CKD, end-stage renal failure, and all-cause mortality in general population [19]. The eGFR-CKD-EPI is an extensively used equation for estimating GFR [20]. The decline of eGFR-CKD-EPI was associated with renal hyperfiltration and impaired GFR in DM patients [21]. These supported the results in our study, which revealed that the eGFR-CKD-EPI was a predictor for the mortality of DN patients within 2 years. Patients with rapid decline of eGFR-CKD-EPI should be brought to the forefront and special treatments should be provided to prevent the mortality of DN patients. DN was associated with higher Scr levels in patients, and high Scr levels indicated a declining renal function [22, 23]. This allied with the results in this study, which indicated that the Scr level was an important predictor for the mortality of elderly DN patients within 2 years. Clinicians should pay special attention to DN patients with high level of Scr. SpO_2_ is an index for oxygenation status of people and tissue hypoxia is an important contributor to diabetic complications [24]. Frequent abnormal blood oxygen in patients was reported to be associated with elevated inflammation in patients [25]. Herein, SpO_2_ was a predictor for the mortality of elderly DN patients within 2 years. In this study, RDW was another predictor for the mortality of elderly DN patients within 2 years. This was supported by several previous studies. Zhang et al. [26] identified that patients with DN were found to be with high level of RDW and RDW was associated with increased risk of progression to ESRD in patients with DN [26]. Another study also demonstrated that high level of RDW was an indicator of prognosis in DN patients and high level of RDW in T2D patients indicated a poor prognosis for DN [27]. SAPS-II is an indicator evaluating the outcomes of patients in ICUs and estimating their risk of mortality [28]. SAPS-II has good power to predict the deaths in ICU, which has been recommended for the identification and mortality prognostication of patients in ICUs [29]. In our study, SAPS-II was found to be a predictor for the mortality in ICU patients with DN. High-risk patients were associated with longer LOS in ICUs and with higher hospital mortality [30]. The prolonged LOS in ICUs has been reported to be a risk factor for infections, which might also increase the risk of death in patients [31]. These gave evidence to the findings in this study, showing that LOS in ICUs was a predictor for the mortality of DN patients in ICUs.

Several limitations existed in our study. Firstly, this study extracted the data from MIMIC-III database, which lacked several important variables including the medications of DN patients, as well as the control of blood glucose of the subjects, and these were closely associated with the outcomes of these patients. Secondly, external validation of the predictive value of the model was not performed. In the future, studies with large scale of sample size were required to validate the findings in our study. Currently, there were numerous machine learning algorithms that can be used for predicting the mortality of elderly patients with DN. Some recent studies have also used principal component analysis- (PCA-) firefly based deep learning model for predicting the occurrence or the detection of diabetic retinopathy [32–34]. The predictive accuracy was evidently improved using these methods. Diabetic nephropathy and DN are common microvascular complications of diabetes mellitus. In our study, we only used logistic regression model, and, in the future, PCA-firefly based deep learning model might be applied in our further studies to improve the predictive ability for the mortality of DN patients and achieve a better tool for the clinicians to quickly and accurately identify those with high risk of death.

## 5. Conclusion

This study established a prediction model for the mortality of DN patients within 2 years based on LOS, temperature, heart rate, SpO_2_, Scr, RDW, SAPS-II, hyperlipidemia, and eGFR-CKD-EPI. The model had good predictive value for the mortality of elderly patients with DN within 2 years. In addition, the model showed good predictive values for female DN patients, DN patients ≤75 years, and DN patients accompanied with CKD.

## Figures and Tables

**Table 1:** Comparisons of the characteristics of surviving and dead patients.

Variables	Total (*n* = 511)	Group			
		Survival group (*n* = 295)	Death group (*n* = 216)	Statistics	*P*
Gender, *n* (%)				*χ * ^2^ = 3.000	0.083
Male	292 (57.14)	159 (53.90)	133 (61.57)		
Female	219 (42.86)	136 (46.10)	83 (38.43)		

Marital status, *n* (%)				*χ * ^2^ = 9.472	0.092
Divorced	34 (6.65)	22 (7.46)	12 (5.56)		
Married	247 (48.34)	143 (48.47)	104 (48.15)		
Separated	3 (0.59)	0 (0.00)	3 (1.39)		
Single	85 (16.63)	53 (17.97)	32 (14.81)		
Unknown	14 (2.74)	5 (1.69)	9 (4.17)		
Widowed	128 (25.05)	72 (24.41)	56 (25.93)		

Ethnicity, *n* (%)				*χ * ^2^ = 9.861	0.079
Asian	18 (3.52)	7 (2.37)	11 (5.09)		
Black	93 (18.20)	63 (21.36)	30 (13.89)		
Hispanic or Latino	12 (2.35)	7 (2.37)	5 (2.31)		
Others	11 (2.15)	8 (2.71)	3 (1.39)		
Unknown	43 (8.41)	20 (6.78)	23 (10.65)		
White	334 (65.36)	190 (64.41)	144 (66.67)		

LOS, *M* (*Q*_1_, *Q*_3_)	2.60 (1.37, 4.79)	2.15 (1.24, 3.84)	3.24 (1.64, 6.93)	*Z* = 4.748	<0.001
Age, *M* (*Q*_1_, *Q*_3_)	74.39 (69.69, 80.12)	73.90 (69.53, 80.02)	75.14 (70.13, 80.31)	*Z* = 1.343	0.179
Respiratory rate, mean ± SD	19.08 ± 5.76	18.66 ± 5.36	19.66 ± 6.23	*t* = −1.90	0.058
Temperature, mean ± SD	36.46 ± 0.95	36.54 ± 0.91	36.36 ± 1.00	*t* = 2.10	0.036
Heart rate, mean ± SD	82.59 ± 17.99	80.94 ± 17.64	84.84 ± 18.25	*t* = −2.43	0.015
SBP, mean ± SD	126.43 ± 28.02	127.96 ± 28.29	124.35 ± 27.58	*t* = 1.44	0.150
DBP, mean ± SD	58.35 ± 16.25	58.07 ± 15.74	58.73 ± 16.96	*t* = −0.45	0.650
MAP, mean ± SD	78.60 ± 18.90	78.16 ± 17.55	79.20 ± 20.63	*t* = −0.60	0.551
SpO_2_, mean ± SD	96.98 ± 4.73	96.90 ± 5.14	97.10 ± 4.10	*t* = −0.50	0.620
WBC, *M* (*Q*_1_, *Q*_3_)	9.70 (7.30, 12.70)	9.60 (7.20, 12.70)	9.70 (7.40, 12.65)	*Z* = 0.488	0.626
RBC, mean ± SD	3.65 ± 0.71	3.69 ± 0.75	3.60 ± 0.65	*t* = 1.37	0.171
Sodium, mean ± SD	137.70 ± 4.71	137.60 ± 4.75	137.83 ± 4.66	*t* = −0.54	0.586
Potassium, mean ± SD	4.64 ± 0.97	4.67 ± 1.00	4.60 ± 0.93	*t* = 0.80	0.426
Phosphate, *M* (*Q*_1_,*Q*_3_)	4.00 (3.30, 4.90)	3.90 (3.20, 4.70)	4.10 (3.30, 5.10)	*Z* = 1.918	0.055
Calcium, mean ± SD	8.69 ± 0.95	8.78 ± 0.95	8.57 ± 0.93	*t* = 2.45	0.014
PLT, *M* (*Q*_1_, *Q*_3_)	216.00 (169.00, 288.00)	218.00 (173.00, 277.00)	208.50 (166.50, 295.00)	*Z* = −0.609	0.542
Lactate, *M* (*Q*_1_, *Q*_3_)	1.60 (1.22, 2.20)	1.60 (1.20, 2.20)	1.70 (1.30, 2.38)	*Z* = 1.750	0.080
INR, *M* (*Q*_1_,*Q*_3_)	1.20 (1.10,1.50)	1.20 (1.10,1.40)	1.30 (1.10,1.50)	*Z* = 2.904	0.004
MCV, mean ± SD	90.89 ± 7.56	90.67 ± 7.72	91.20 ± 7.34	*t* = −0.78	0.436
Magnesium, mean ± SD	2.05 ± 0.45	2.05 ± 0.44	2.05 ± 0.46	*t* = 0.01	0.989
Glucose, *M* (*Q*_1_, *Q*_3_)	166.00 (125.00, 242.00)	176.00 (130.00, 249.00)	158.50 (119.50, 229.00)	*Z* = −1.983	0.047
Creatinine, *M* (*Q*_1_, *Q*_3_)	2.70 (1.70, 4.30)	2.40 (1.60, 4.10)	2.90 (1.90, 4.45)	*Z* = 2.571	0.010
BUN, *M* (*Q*_1_, *Q*_3_)	45.00 (31.00, 68.00)	44.00 (30.00, 65.00)	48.00 (32.00, 71.00)	*Z* = 2.022	0.043
Bicarbonate, mean ± SD	24.37 ± 5.39	24.06 ± 4.99	24.79 ± 5.88	*t* = −1.47	0.142
Hematocrit, mean ± SD	32.91 ± 6.02	33.11 ± 6.42	32.64 ± 5.43	*t* = 0.89	0.374
Hemoglobin, mean ± SD	10.81 ± 1.95	10.95 ± 2.07	10.62 ± 1.77	*t* = 1.97	0.049
MCHC, mean ± SD	32.85 ± 1.60	33.06 ± 1.56	32.56 ± 1.61	*t* = 3.55	<0.001
RDW, mean ± SD	15.81 ± 1.89	15.38 ± 1.72	16.39 ± 1.95	*t* = −6.22	<0.001
COPD, *n* (%)				*χ * ^2^ = 0.526	0.468
No	419 (82.00)	245 (83.05)	174 (80.56)		
Yes	92 (18.00)	50 (16.95)	42 (19.44)		

AF, *n* (%)				*χ * ^2^ = 1.546	0.214
No	286 (55.97)	172 (58.31)	114 (52.78)		
Yes	225 (44.03)	123 (41.69)	102 (47.22)		

Liver cirrhosis, *n* (%)				*χ * ^2^ = 0.097	0.755
No	488 (95.50)	281 (95.25)	207 (95.83)		
Yes	23 (4.50)	14 (4.75)	9 (4.17)		

Respiratory failure, *n* (%)				*χ * ^2^ = 13.735	<0.001
No	355 (69.47)	224 (75.93)	131 (60.65)		
Yes	156 (30.53)	71 (24.07)	85 (39.35)		

Hyperlipidemia, *n* (%)				*χ * ^2^ = 27.292	<0.001
No	267 (52.25)	125 (42.37)	142 (65.74)		
Yes	244 (47.75)	170 (57.63)	74 (34.26)		

Malignant cancer, *n* (%)				*χ * ^2^ = 0.070	0.792
No	405 (79.26)	235 (79.66)	170 (78.70)		
Yes	106 (20.74)	60 (20.34)	46 (21.30)		

SAPS-II score, mean ± SD	42.79 ± 11.78	40.74 ± 11.18	45.60 ± 12.02	*t* = −4.70	<0.001
SOFA score, *M* (*Q*_1_, *Q*_3_)	6.00 (4.00, 8.00)	5.00 (3.00, 7.00)	6.00 (4.00, 8.00)	*Z* = 4.448	<0.001
Insulin, *n* (%)				*χ * ^2^ = 4.861	0.027
No	33 (6.46)	13 (4.41)	20 (9.26)		
Yes	478 (93.54)	282 (95.59)	196 (90.74)		

Metformin, *n* (%)				*χ * ^2^ = 0.254	0.615
No	497 (97.26)	286 (96.95)	211 (97.69)		
Yes	14 (2.74)	9 (3.05)	5 (2.31)		

Survival time, *M* (*Q*_1_, *Q*_3_)	652.00 (87.00, 3650.00)	3650.00 (1088.00, 3650.00)	61.50 (17.00, 165.50)	*Z* = −19.702	<0.001
eGFR-MDRD, *M* (*Q*_1_, *Q*_3_)	21.80 (11.97, 34.05)	23.02 (12.55, 37.12)	19.63 (11.32, 30.85)	*Z* = −2.626	0.009
eGFR-CKD-EPI, *M* (*Q*_1_, *Q*_3_)	21.44 (12.93, 33.35)	23.94 (13.67, 36.22)	19.51 (11.86, 29.22)	*Z* = −3.189	0.001
CVD, *n* (%)				*χ * ^2^ = 2.528	0.112
No	122 (23.87)	78 (26.44)	44 (20.37)		
Yes	389 (76.13)	217 (73.56)	172 (79.63)		

CKD, *n* (%)				*χ * ^2^ = 8.774	0.003
No	178 (34.83)	87 (29.49)	91 (42.13)		
Yes	333 (65.17)	208 (70.51)	125 (57.87)		

Myocardial infarction, *n* (%)				*χ * ^2^ = 0.027	0.870
No	341 (66.73)	196 (66.44)	145 (67.13)		
Yes	170 (33.27)	99 (33.56)	71 (32.87)		

Hypertension, *n* (%)				*χ * ^2^ = 1.142	0.285
No	400 (78.28)	226 (76.61)	174 (80.56)		
Yes	111 (21.72)	69 (23.39)	42 (19.44)		

Peripheral vascular disease, *n* (%)				*χ * ^2^ = 4.106	0.043
No	481 (94.13)	283 (95.93)	198 (91.67)		
Yes	30 (5.87)	12 (4.07)	18 (8.33)		

Survival time within 2 years, *M* (*Q*_1_,*Q*_3_)	730.00 (87.00, 730.00)	730.00 (730.00, 730.00)	61.50 (17.00, 165.50)	*Z* = −21.501	<0.001
Death within 10 years, *n* (%)				*χ * ^2^ = 189.837	<0.001
No	172 (33.66)	172 (58.31)	0 (0.00)		
Yes	339 (66.34)	123 (41.69)	216 (100.00)		

**Table 2:** Baseline data of the participants in the training set and the testing set.

Variable	Total (*n* = 511)	Testing set (*n* = 153)	Training set (*n* = 358)	Statistical magnitude	*P*
Gender, *n* (%)				*χ * ^2^ = 0.012	0.911
Male	292 (57.14)	88 (57.52)	204 (56.98)		
Female	219 (42.86)	65 (42.48)	154 (43.02)		

Marital status, *n* (%)				*χ * ^2^ = 5.188	0.393
Divorced	34 (6.65)	10 (6.54)	24 (6.70)		
Married	247 (48.34)	73 (47.71)	174 (48.60)		
Separated	3 (0.59)	0 (0.00)	3 (0.84)		
Single	85 (16.63)	32 (20.92)	53 (14.80)		
Unknown	14 (2.74)	4 (2.61)	10 (2.79)		
Widowed	128 (25.05)	34 (22.22)	94 (26.26)		

Ethnicity, *n* (%)				*χ * ^2^ = 3.443	0.632
Asian	18 (3.52)	3 (1.96)	15 (4.19)		
Black	93 (18.20)	28 (18.30)	65 (18.16)		
Hispanic or Latino	12 (2.35)	5 (3.27)	7 (1.96)		
Others	11 (2.15)	2 (1.31)	9 (2.51)		
Unknown	43 (8.41)	12 (7.84)	31 (8.66)		
White	334 (65.36)	103 (67.32)	231 (64.53)		

LOS, *M* (*Q*_1_, *Q*_3_)	2.60 (1.37, 4.79)	2.93 (1.41, 5.02)	2.41 (1.35, 4.38)	*Z* = 1.135	0.256
Age, *M* (*Q*_1_, *Q*_3_)	74.39 (69.69, 80.12)	73.98 (69.44, 79.66)	74.60 (69.92, 80.30)	*Z* = −0.983	0.326
Respiratory rate, mean ± SD	19.08 ± 5.76	18.76 ± 6.00	19.22 ± 5.66	*t* = −0.84	0.404
Temperature, mean ± SD	36.46 ± 0.95	36.35 ± 0.97	36.51 ± 0.94	*t* = −1.74	0.083
Heart rate, mean ± SD	82.59 ± 17.99	82.61 ± 17.29	82.58 ± 18.30	*t* = 0.01	0.989
SBP, mean ± SD	126.43 ± 28.02	126.34 ± 29.08	126.47 ± 27.60	*t* = −0.05	0.962
DBP, mean ± SD	58.35 ± 16.25	57.85 ± 13.00	58.56 ± 17.48	*t* = −0.51	0.611
MAP, mean ± SD	78.60 ± 18.90	79.54 ± 16.45	78.19 ± 19.87	*t* = 0.79	0.428
SpO_2_, mean ± SD	96.98 ± 4.73	97.16 ± 3.84	96.91 ± 5.06	*t* = 0.62	0.534
WBC, *M* (*Q*_1_, *Q*_3_)	9.70 (7.30, 12.70)	9.40 (7.00, 12.00)	9.70 (7.40, 12.70)	*Z* = −1.160	0.246
RBC, mean ± SD	3.65 ± 0.71	3.62 ± 0.74	3.66 ± 0.70	*t* = −0.59	0.555
Sodium, mean ± SD	137.70 ± 4.71	138.10 ± 4.17	137.53 ± 4.92	*t* = 1.33	0.183
Potassium, mean ± SD	4.64 ± 0.97	4.67 ± 0.96	4.64 ± 0.98	*t* = 0.34	0.737
Phosphate, *M* (*Q*_1_, *Q*_3_)	4.00 (3.30, 4.90)	4.00 (3.30, 4.70)	4.00 (3.30, 4.90)	*Z* = −0.253	0.800
Calcium, mean ± SD	8.69 ± 0.95	8.66 ± 0.96	8.71 ± 0.94	*t* = −0.58	0.562
PLT, *M* (*Q*_1_, *Q*_3_)	216.00 (169.00, 288.00)	208.00 (159.00, 269.00)	218.50 (173.00, 289.00)	*Z* = −1.352	0.176
Lactate, *M* (*Q*_1_,*Q*_3_)	1.60 (1.22, 2.20)	1.60 (1.20, 2.30)	1.60 (1.26, 2.20)	*Z* = 0.520	0.603
INR, *M* (*Q*_1_,*Q*_3_)	1.20 (1.10, 1.50)	1.20 (1.10, 1.50)	1.20 (1.10, 1.40)	*Z* = 0.507	0.612
MCV, mean ± SD	90.89 ± 7.56	91.00 ± 7.42	90.85 ± 7.63	*t* = 0.20	0.838
Magnesium, mean ± SD	2.05 ± 0.45	2.08 ± 0.52	2.04 ± 0.42	*t* = 0.97	0.334
Glucose, *M* (*Q*_1_, *Q*_3_)	166.00 (125.00, 242.00)	162.00 (124.00, 230.00)	168.50 (125.00, 249.00)	*Z* = −0.668	0.504
Creatinine, *M* (*Q*_1_, *Q*_3_)	2.70 (1.70, 4.30)	2.80 (1.80, 4.40)	2.65 (1.70, 4.30)	*Z* = 1.156	0.248
BUN, *M* (*Q*_1_, *Q*_3_)	45.00 (31.00, 68.00)	42.00 (32.00, 69.00)	46.00 (31.00, 68.00)	*Z* = −0.179	0.858
Bicarbonate, mean ± SD	24.37 ± 5.39	24.41 ± 5.25	24.36 ± 5.46	*t* = 0.09	0.927
Hematocrit, mean ± SD	32.91 ± 6.02	32.63 ± 6.23	33.03 ± 5.93	*t* = −0.69	0.493
Hemoglobin, mean ± SD	10.81 ± 1.95	10.73 ± 2.02	10.84 ± 1.93	*t* = −0.62	0.538
MCHC, mean ± SD	32.85 ± 1.60	32.84 ± 1.66	32.85 ± 1.58	*t* = −0.09	0.932
RDW, mean ± SD	15.81 ± 1.89	15.77 ± 1.74	15.82 ± 1.95	*t* = −0.28	0.782
COPD, *n* (%)				*χ * ^2^ = 1.254	0.263
No	419 (82.00)	121 (79.08)	298 (83.24)		
Yes	92 (18.00)	32 (20.92)	60 (16.76)		

AF, *n* (%)				*χ * ^2^ = 1.665	0.197
No	286 (55.97)	79 (51.63)	207 (57.82)		
Yes	225 (44.03)	74 (48.37)	151 (42.18)		

Liver cirrhosis, *n* (%)				*χ * ^2^ = 0.269	0.604
No	488 (95.50)	145 (94.77)	343 (95.81)		
Yes	23 (4.50)	8 (5.23)	15 (4.19)		

Respiratory failure, *n* (%)				*χ * ^2^ = 3.798	0.051
No	355 (69.47)	97 (63.40)	258 (72.07)		
Yes	156 (30.53)	56 (36.60)	100 (27.93)		

Hyperlipidemia, *n* (%)				*χ * ^2^ = 0.956	0.328
No	267 (52.25)	85 (55.56)	182 (50.84)		
Yes	244 (47.75)	68 (44.44)	176 (49.16)		

Malignant cancer, *n* (%)				*χ * ^2^ = 1.571	0.210
No	405 (79.26)	116 (75.82)	289 (80.73)		
Yes	106 (20.74)	37 (24.18)	69 (19.27)		

SAPS-II score, mean ± SD	42.79 ± 11.78	43.12 ± 12.47	42.66 ± 11.48	*t* = 0.41	0.686
SOFA score, *M* (*Q*_1_, *Q*_3_)	6.00 (4.00, 8.00)	6.00 (4.00, 8.00)	5.00 (4.00, 7.00)	*Z* = 2.131	0.033
Insulin, *n* (%)				*χ * ^2^ = 2.326	0.127
No	33 (6.46)	6 (3.92)	27 (7.54)		
Yes	478 (93.54)	147 (96.08)	331 (92.46)		

Metformin, *n* (%)				Fisher	0.768
No	497 (97.26)	148 (96.73)	349 (97.49)		
Yes	14 (2.74)	5 (3.27)	9 (2.51)		

Survival time, *M* (*Q*_1_, *Q*_3_)	652.00 (87.00, 3650.00)	770.00 (103.00, 3650.00)	584.00 (80.00, 3650.00)	*Z* = 0.813	0.416
eGFR-MDRD, *M* (*Q*_1_, *Q*_3_)	21.80 (11.97, 34.05)	18.92 (11.86, 32.84)	22.16 (12.12, 34.48)	*Z* = −1.191	0.234
eGFR-CKD-EPI, *M* (*Q*_1_, *Q*_3_)	21.44 (12.93, 33.35)	20.47 (11.85, 32.05)	21.98 (13.41, 33.94)	*Z* = -1.418	0.156
CVD, *n* (%)				*χ * ^2^ = 0.328	0.567
No	122 (23.87)	34 (22.22)	88 (24.58)		
Yes	389 (76.13)	119 (77.78)	270 (75.42)		

CKD, *n* (%)				*χ * ^2^ = 0.564	0.453
No	178 (34.83)	57 (37.25)	121 (33.80)		
Yes	333 (65.17)	96 (62.75)	237 (66.20)		

Myocardial infarction, *n* (%)				*χ * ^2^ = 0.185	0.667
No	341 (66.73)	100 (65.36)	241 (67.32)		
Yes	170 (33.27)	53 (34.64)	117 (32.68)		

Hypertension, *n* (%)				*χ * ^2^ = 0.274	0.601
No	400 (78.28)	122 (79.74)	278 (77.65)		
Yes	111 (21.72)	31 (20.26)	80 (22.35)		

Peripheral vascular disease, *n* (%)				*χ * ^2^ = 0.000	0.994
No	481 (94.13)	144 (94.12)	337 (94.13)		
Yes	30 (5.87)	9 (5.88)	21 (5.87)		

Survival time within 2 years, *M* (*Q*_1_, *Q*_3_)	730.00(87.00, 730.00)	730.00(103.00, 730.00)	730.00(80.00, 730.00)	*Z* = 0.964	0.335
Death within 2 years, *n* (%)				*χ * ^2^ = 0.516	0.473
No	295 (57.73)	92 (60.13)	203 (56.70)		
Yes	216 (42.27)	61 (39.87)	155 (43.30)		

LOS: length of stay, SBP: systolic blood pressure, DBP: diastolic blood pressure, MAP: mean arterial pressure, SpO_2_: peripheral oxygen saturation, WBC: white blood cells, RBC: red blood cells, INR: international normalized ratio, MCV: mean corpuscular volume, MCHC: mean corpuscular hemoglobin concentration, RDW: red cell distribution width, COPD: chronic obstructive pulmonary disease, AF: atrial fibrillation, eGFR-CKD-EPI: the Chronic Kidney Disease Epidemiology Collaboration equation for estimated glomerular filtration rate, eGFR-MDRD: the Modification of Diet in Renal Disease equation for estimated glomerular filtration rate, CKD: chronic kidney disease, CVD: cardiovascular diseases, SOFA: Sequential Organ Failure Assessment, SAPS-II: the simplified acute physiology score-II.

**Table 3:** Comparisons between the characteristics of patients in the survival group and death group in the training set.

Variable	Survival within 2 years (*n* = 203)	Death within 2 years (*n* = 155)	Statistical magnitude	*P*
Gender, *n* (%)			*χ * ^2^ = 1.495	0.221
Male	110 (54.19)	94 (60.65)		
Female	93 (45.81)	61 (39.35)		

Marital status, *n* (%)			*χ * ^2^ = 10.722	0.057
Divorced	17 (8.37)	7 (4.52)		
Married	98 (48.28)	76 (49.03)		
Separated	0 (0.00)	3 (1.94)		
Single	33 (16.26)	20 (12.90)		
Unknown	3 (1.48)	7 (4.52)		
Widowed	52 (25.62)	42 (27.10)		

Ethnicity, *n* (%)			Fisher	0.134
Asian	6 (2.96)	9 (5.81)		
Black	44 (21.67)	21 (13.55)		
Hispanic or Latino	4 (1.97)	3 (1.94)		
Others	7 (3.45)	2 (1.29)		
Unknown	14 (6.90)	17 (10.97)		
White	128 (63.05)	103 (66.45)		

LOS, *M* (*Q*_1_, *Q*_3_)	2.15 (1.22, 3.66)	3.01 (1.61, 6.50)	*Z* = 3.734	<0.001
Age, *M* (*Q*_1_, *Q*_3_)	73.59 (69.32, 80.24)	76.03 (70.54, 80.87)	*Z* = 1.770	0.077
Respiratory rate, mean ± SD	18.87 ± 5.16	19.69 ± 6.23	*t* = −1.33	0.185
Temperature, mean ± SD	36.59 ± 0.87	36.41 ± 1.02	*t* = 1.75	0.081
Heart rate, mean ± SD	80.12 ± 17.07	85.81 ± 19.38	*t* = −2.95	0.003
SBP, mean ± SD	127.97 ± 27.43	124.51 ± 27.80	*t* = 1.18	0.240
DBP, mean ± SD	58.17 ± 16.54	59.08 ± 18.68	*t* = −0.49	0.625
MAP, mean ± SD	77.33 ± 17.95	79.32 ± 22.14	*t* = −0.91	0.363
SpO_2_, mean ± SD	96.52 ± 5.86	97.42 ± 3.73	*t* = −1.77	0.077
WBC, *M* (*Q*_1_, *Q*_3_)	9.50 (7.30, 12.40)	10.20 (7.50, 13.00)	*Z* = 1.211	0.226
RBC, mean ± SD	3.72 ± 0.74	3.58 ± 0.64	*t* = 1.85	0.064
Sodium, mean ± SD	137.45 ± 4.91	137.63 ± 4.94	*t* = −0.34	0.733
Potassium, mean ± SD	4.63 ± 1.00	4.64 ± 0.95	*t* = −0.03	0.979
Phosphate, *M* (*Q*_1_, *Q*_3_)	3.90 (3.20, 4.80)	4.00 (3.40, 5.10)	*Z* = 1.506	0.132
Calcium, mean ± SD	8.82 ± 0.96	8.57 ± 0.91	*t* = 2.48	0.014
PLT, *M* (*Q*_1_, *Q*_3_)	218.00 (174.00, 273.00)	220.00 (170.00, 303.00)	*Z* = 0.113	0.910
Lactate, *M* (*Q*_1_, *Q*_3_)	1.58 (1.20, 2.10)	1.70 (1.30, 2.30)	*Z* = 1.454	0.146
INR, *M* (*Q*_1_, *Q*_3_)	1.20 (1.10,1.40)	1.30 (1.10,1.60)	*Z* = 2.767	0.006
MCV, mean ± SD	90.36 ± 7.57	91.48 ± 7.67	*t* = −1.38	0.167
Magnesium, mean ± SD	2.03 ± 0.38	2.05 ± 0.46	*t* = −0.39	0.700
Glucose, *M* (*Q*_1_, *Q*_3_)	178.00 (125.00, 253.00)	163.00 (125.00, 239.00)	*Z* = −0.995	0.320
Creatinine, *M* (*Q*_1_, *Q*_3_)	2.30 (1.60, 4.20)	2.90 (1.90, 4.30)	*Z* = 2.100	0.036
BUN, *M* (*Q*_1_,*Q*_3_)	43.00 (30.00,61.00)	50.00 (32.00,72.00)	*Z* = 2.447	0.014
Bicarbonate, mean ± SD	24.20 ± 4.97	24.56 ± 6.05	*t* = −0.60	0.548
Hematocrit, mean ± SD	33.37 ± 6.28	32.59 ± 5.43	*t* = 1.24	0.215
Hemoglobin, mean ± SD	11.02 ± 2.01	10.61 ± 1.79	*t* = 2.03	0.043
MCHC, mean ± SD	33.05 ± 1.56	32.59 ± 1.58	*t* = 2.78	0.006
RDW, mean ± SD	15.40 ± 1.74	16.37 ± 2.07	*t* = −4.71	<0.001
COPD, *n* (%)			*χ * ^2^ = 1.320	0.251
No	173 (85.22)	125 (80.65)		
Yes	30 (14.78)	30 (19.35)		

AF, *n* (%)			*χ * ^2^ = 0.612	0.434
No	121 (59.61)	86 (55.48)		
Yes	82 (40.39)	69 (44.52)		

Liver cirrhosis, *n* (%)			*χ * ^2^ = 0.069	0.792
No	194 (95.57)	149 (96.13)		
Yes	9 (4.43)	6 (3.87)		

Respiratory failure, *n* (%)			*χ * ^2^ = 4.282	0.039
No	155 (76.35)	103 (66.45)		
Yes	48 (23.65)	52 (33.55)		

Hyperlipidemia, *n* (%)			*χ * ^2^ = 24.505	<0.001
No	80 (39.41)	102 (65.81)		
Yes	123 (60.59)	53 (34.19)		

Malignant cancer, *n* (%)			*χ * ^2^ = 0.604	0.437
No	161 (79.31)	128 (82.58)		
Yes	42 (20.69)	27 (17.42)		

SAPS-II score, mean ± SD	40.28 ± 10.74	45.77 ± 11.71	*t* = −4.62	<0.001
SOFA score, *M* (*Q*_1_, *Q*_3_)	5.00 (3.00, 7.00)	6.00 (4.00, 8.00)	*Z* = 4.397	<0.001
Insulin, *n* (%)			*χ * ^2^ = 3.031	0.082
No	11 (5.42)	16 (10.32)		
Yes	192 (94.58)	139 (89.68)		

Metformin, *n* (%)			Fisher	0.309
No	196 (96.55)	153 (98.71)		
Yes	7 (3.45)	2 (1.29)		

eGFR-MDRD, *M* (*Q*_1_, *Q*_3_)	25.42 (12.48, 39.27)	20.41 (11.55, 31.45)	*Z* = −2.266	0.023
eGFR-CKD-EPI, *M* (*Q*_1_, *Q*_3_)	25.60 (13.67, 36.52)	19.68 (12.99, 29.23)	*Z* = −2.705	0.007
CVD, *n* (%)			*χ * ^2^ = 0.590	0.442
No	53 (26.11)	35 (22.58)		
Yes	150 (73.89)	120 (77.42)		

CKD, *n* (%)			*χ * ^2^ = 3.771	0.052
No	60 (29.56)	61 (39.35)		
Yes	143 (70.44)	94 (60.65)		

Myocardial infarction, *n* (%)			*χ * ^2^ = 0.022	0.881
No	136 (67.00)	105 (67.74)		
Yes	67 (33.00)	50 (32.26)		

Hypertension, *n* (%)			*χ * ^2^ = 0.027	0.870
No	157 (77.34)	121 (78.06)		
Yes	46 (22.66)	34 (21.94)		

Diabetic retinopathy, *n* (%)			*χ * ^2^ = 4.888	0.027
No	160 (78.82)	136 (87.74)		
Yes	43 (21.18)	19 (12.26)		

Peripheral vascular disease, *n* (%)			*χ * ^2^ = 0.750	0.386
No	193 (95.07)	144 (92.90)		
Yes	10 (4.93)	11 (7.10)		

LOS: length of stay, SBP: systolic blood pressure, DBP: diastolic blood pressure, MAP: mean arterial pressure, SpO_2_: peripheral oxygen saturation, WBC: white blood cells, RBC: red blood cells, INR: international normalized ratio, MCV: mean corpuscular volume, MCHC: mean corpuscular hemoglobin concentration, RDW: red cell distribution width, COPD: chronic obstructive pulmonary disease, AF: atrial fibrillation, eGFR-CKD-EPI: the Chronic Kidney Disease Epidemiology Collaboration equation for estimated glomerular filtration rate, eGFR-MDRD: the Modification of Diet in Renal Disease equation for estimated glomerular filtration rate, CKD: chronic kidney disease, CVD: cardiovascular diseases, SOFA: Sequential Organ Failure Assessment, SAPS-II: the simplified acute physiology score-II.

**Table 4:** Predictors for mortality of elderly patients with DN.

Character	*β*	SE	*z*	*P* > |z|	OR	OR (lower (95%))	OR (upper (95%))
LOS	0.09	0.03	2.90	0.004	1.10	1.03	1.17
Temperature	−0.29	0.08	−3.57	<0.001	0.74	0.63	0.88
Heart rate	0.03	0.01	3.49	<0.001	1.03	1.01	1.04
SpO_2_	0.05	0.03	2.00	0.046	1.06	1.01	1.11
Creatinine	−0.19	0.09	−2.16	0.031	0.83	0.69	0.98
RDW percent	0.22	0.07	3.36	0.001	1.25	1.10	1.42
SAPS-II	0.02	0.01	1.97	0.049	1.02	1.01	1.05
Hyperlipidemia	−0.84	0.25	−3.40	0.001	0.43	0.27	0.70
eGFR-CKD-EPI	−0.03	0.01	−2.45	0.014	0.97	0.94	0.99

LOS: length of stay, SpO_2_: peripheral oxygen saturation, eGFR-CKD-EPI: the Chronic Kidney Disease Epidemiology Collaboration equation for estimated glomerular filtration rate, SAPS-II: the simplified acute physiology score-II.

**Table 5:** The predictive value of the model.

Data set	Sensitivity (95% CI)	Specificity (95% CI)	PPV (95% CI)	NPV (95% CI)	AUC (95% CI)	Accuracy (95% CI)
Training set	0.85 (0.80–0.91)	0.59 (0.52–0.65)	0.61 (0.55–0.68)	0.84 (0.78–0.90)	0.78 (0.73–0.83)	0.70 (0.65–0.75)
Testing set	0.90 (0.83–0.98)	0.47 (0.37–0.57)	0.53 (0.43–0.62)	0.88 (0.79–0.97)	0.72 (0.63–0.80)	0.64 (0.56–0.72)

CI: confidence interval, AUC: area under the curve, NPV: negative predictive value, PPV: positive predictive value.

**Table 6:** The predictive value of the model in different subgroups.

Subgroup	Sensitivity (95% CI)	Specificity (95% CI)	PPV (95% CI)	NPV (95% CI)	AUC (95% CI)	Accuracy (95% CI)
Gender						
Male	0.90 (0.80–0.99)	0.39 (0.25–0.52)	0.54 (0.42–0.66)	0.83 (0.67–0.98)	0.66 (0.55–0.78)	0.61 (0.51–0.72)
Female	0.91 (0.79–1.00)	0.56 (0.41–0.71)	0.51 (0.36–0.67)	0.92 (0.82–1.00)	0.78 (0.65–0.91)	0.68 (0.56–0.79)

Age						
>75 years	0.88 (0.75–1.00)	0.36 (0.22–0.50)	0.43 (0.30–0.57)	0.84 (0.68–1.00)	0.65 (0.52–0.78)	0.54 (0.43–0.66)
≤75 years	0.92 (0.83–1.00)	0.57 (0.43–0.72)	0.62 (0.49–0.75)	0.90 (0.79–1.00)	0.78 (0.68–0.88)	0.72 (0.63–0.82)

CKD						
Yes	0.90 (0.80–1.00)	0.51 (0.39–0.63)	0.47 (0.34–0.59)	0.92 (0.83–1.00)	0.74 (0.64–0.84)	0.64 (0.54–0.73)
No	0.90 (0.79–1.00)	0.37 (0.19–0.55)	0.61 (0.47–0.76)	0.77 (0.54–1.00)	0.67 (0.52–0.82)	0.65 (0.53–0.77)

CVD						
Yes	0.90 (0.82–0.98)	0.46 (0.34–0.58)	0.57 (0.46–0.67)	0.86 (0.75–0.97)	0.71 (0.61–0.80)	0.66 (0.57–0.74)
No	0.89 (0.68–1.00)	0.48 (0.28–0.68)	0.38 (0.17–0.59)	0.92 (0.78–1.00)	0.71 (0.50–0.92)	0.59 (0.42–0.75)

CI: confidence interval, AUC: area under the curve, NPV: negative predictive value, PPV: positive predictive value, CKD: chronic kidney disease, CVD: cardiovascular diseases.

## Data Availability

The data used to support the findings of this study can be obtained from the corresponding author upon request.

## Supplementary Materials

Supplementary Materials Supplementary Table 1: sensitivity analysis of the data before and after the manipulation of the missing value.
