# Supplementary material for: Construction of a Prediction Model for the Mortality of Elderly Patients with Diabetic Nephropathy
Source: J Healthc Eng. 2022 Sep 12;2022:5724050. doi: 10.1155/2022/5724050 (PMC9484980; doi:10.1155/2022/5724050)
Supplement: Supplementary Materials — Supplementary Table 1: sensitivity analysis of the data before and after the manipulation of the missing value. [file 5724050.f1.docx]

Supplementary Table 1 sensitivity analysis of the data before and after the manipulation of the missing value

| Variable | Missing data (%) | After manipulation (n=511) | Before manipulation (n=511) | Statistical magnitude | *P* |
| --- | --- | --- | --- | --- | --- |
| Respiratory Rate, Mean±SD | 12 (2.35) | 18.57±5.69 | 18.57±5.72 | t=0.04 | 0.972 |
| Temperature, Mean±SD | 12 (2.35) | 36.56±0.95 | 36.56±0.95 | t=0.09 | 0.926 |
| Heart Rate, Mean±SD | 12 (2.35) | 85.26±18.09 | 85.20±18.16 | t=0.08 | 0.939 |
| SBP, Mean±SD | 12 (2.35) | 129.83±29.14 | 129.66±29.24 | t=0.13 | 0.897 |
| DBP, Mean±SD | 12 (2.35) | 62.22±17.91 | 62.14±17.97 | t=0.10 | 0.918 |
| MAP, Mean±SD | 12 (2.35) | 82.19±19.83 | 82.12±19.92 | t=0.08 | 0.937 |
| SPO_2_, Mean±SD | 12 (2.35) | 97.05±6.06 | 97.06±6.06 | t=-0.05 | 0.960 |
| Phosphate, M (Q_1_,Q_3_) | 6 (1.17) | 4.10 (3.30,5.30) | 4.10 (3.30,5.30) | Z=0.01 | 0.993 |
| Calcium, Mean±SD | 4 (0.78) | 8.72±0.97 | 8.72±0.97 | t=-0.03 | 0.978 |

SBP: systolic blood pressure, DBP: diastolic blood pressure, MAP: mean arterial pressure, SpO2: peripheral oxygen saturation
